# Recent Advances in Tumor Targeting via EPR Effect for Cancer Treatment

**DOI:** 10.3390/jpm11060571

**Published:** 2021-06-18

**Authors:** Md Abdus Subhan, Satya Siva Kishan Yalamarty, Nina Filipczak, Farzana Parveen, Vladimir P. Torchilin

**Affiliations:** 1 Department of Chemistry, Shah Jalal University of Science and Technology, Sylhet 3114, Bangladesh; 2 CPBN, Department of Pharmaceutical Sciences, Northeastern University, Boston, MA 02115, USA; yalamarty.s@northeastern.edu (S.S.K.Y.); nina.filipczak@gmail.com (N.F.); farzanaparveenphd@gmail.com (F.P.); 3 Department of Pharmaceutics, Faculty of Pharmacy, The Islamia University of Bahawalpur, Punjab 63100, Pakistan; 4 Department of Oncology, Radiotherapy and Plastic Surgery, I.M. Sechenov First Moscow State Medical University (Sechenov University), 119991 Moscow, Russia

**Keywords:** EPR-based therapy, passive targeting, heterogeneity, solid-tumor, EPR-imaging techniques

## Abstract

Cancer causes the second-highest rate of death world-wide. A major shortcoming inherent in most of anticancer drugs is their lack of tumor selectivity. Nanodrugs for cancer therapy administered intravenously escape renal clearance, are unable to penetrate through tight endothelial junctions of normal blood vessels and remain at a high level in plasma. Over time, the concentration of nanodrugs builds up in tumors due to the EPR effect, reaching several times higher than that of plasma due to the lack of lymphatic drainage. This review will address in detail the progress and prospects of tumor-targeting via EPR effect for cancer therapy.

## 1. Introduction

The EPR effect was first discovered by Maeda and colleagues in solid murine tumors [1,2]. The polymer-drug conjugates were i.v. administered, and 10-to-100-fold higher concentrations were achieved relative to free drug administration [2,3,4]. Passively targeted cancer drugs at first reached the clinic about 30 years ago with the approval of an EPR-based drug, a PEGylated liposomal drug, DOXIL. Nanocarriers preferentially accumulate in the tumor through passive targeting due to a leaky vasculature and defective lymphatic drainage in solid tumors. The permeability of a chaotic vasculature and tumor microenvironment (TME) and retention can lead to the accumulation of macromolecules in TME by 70-fold. The leaky and defective vasculature created due to the rapid vascularization vital to the support of malignant tumors, coupled with imperfect lymphatic drainage, allows the EPR effect. The tumor vasculature is leaky and also irregular in diameter, shape, and density with discontinuous vessels. This results in several conditions, including heterogenous perfusion in the tumor, elevated interstitial fluid pressure from the extravasation of fluid, hypoxia, and an acidic environment [5]. EPR-based drug delivery depends on various factors, including circulation time, targeting, and the ability to overcome barriers, which are dependent on size, shape, and surface properties of the drug particles. Passive-targeting is mainly based on a diffusion mechanism. As a result, size is a crucial factor in the EPR-dependent delivery process. Studies have indicated that a nanoparticle size range of approximately 40 to 400 nm is suitable to ensure long circulation time, and enhanced accumulation in tumors with reduced renal clearance [6]. Shape and morphology also play important roles in passive targeting. Generally, rigid, spherical particles of size ranging from 50 to 200 nm have the highest tendency to long circulation, to avoid uptake by liver and spleen, but large enough to avoid kidney clearance [7,8]. Surface properties play a critical role in determining the internalization process of the drug particles into the target cell. To avoid opsonization and subsequent clearance by the RES, surface modification of polymers using PEG can be effective to a certain extent. Thus, the EPR-based drug delivery can be modulated by modifying the size, shape and sometimes by surface alteration of the nanoparticles.

Currently, a number of passively targeted nanoparticles are in clinical use including, Abraxene, Doxil, Marqibo, Myocet, and DuanoXome. Many other nanoparticles have shown promising therapeutic efficacy in clinical trials.

Major drawbacks of passive targeting include the inability to distinguish between healthy and diseased tissues, inadequate tumor accumulation, intra- and inter tumoral as well as inter-individual tumor heterogeneity. Different vascular and TME parameters contribute to the heterogeneity in EPR-mediated nanoparticle accumulation. These include vascular permeability, endothelial cell receptor expression, and vascular maturation at the vessel level. Several stromal parameters, including extracellular matrix (ECM), tumor cell density, hypoxia, and interstitial fluid pressure, contribute to heterogeneity in EPR-based tumor targeting responses. All of these pathophysiological parameters are factors necessary to be taken into consideration for the development of personalized and improved nanodrug treatments using the EPR effect. The extent of tumor accumulation always varies between tumor types, and in patients, making it necessary to determine the EPR effect. Thus, the application of direct and indirect imaging and other technologies is necessary to evaluate the degree of the EPR-effect. The presence of an EPR and non-EPR tumor in the EPR and non-EPR patients may help improve the EPR-based drug delivery systems for success in the clinic.

Dense cancer stroma is a critical component of the TME, where it has crucial roles in tumor initiation, progression, and metastasis. Most anticancer therapies target cancer cells specifically. However, tumor stromal factors can promote resistance of cancer cells to such therapies, ultimately resulting in deadly diseases such as PDAC (pancreatic ductal carcinoma). Therefore, novel anticancer therapies should be a combination of anticancer and anti-stroma therapeutic agents [9]. Approaches have been made to enhance the EPR-targeted drug accumulation to the tumor while considering cancer stromal barriers. For instance, in the use of the ADC (antibody-drug conjugate) drugs with a scaffold for cancer (CA) stromal (S) targeting (T) (CAST) [10]. In CAST therapy, stroma-targeting immunoconjugates bound to the stroma generate a scaffold, from which controlled release of cytotoxic drugs occurred and afterward diffused throughout the tumor tissue to damage both tumor cells and tumor vessels. The CAST therapy was thus reported as a new mode of cancer therapy, especially for refractory, stromal-rich cancers. Since the first CAST therapy was reported over 10 years ago, there have been several appreciated experimental studies and review works supporting and promoting CAST therapy [11,12,13,14,15,16,17,18].

Several strategies to overcome heterogeneity in EPR-based tumor accumulation can be employed to improve nanoparticle-based cancer treatments, including enhancing, combining, bypassing, and imaging. Enhancing pharmacological and physical means such as radiotherapy, hyperthermia, and sonoporation can be used to enhance the EPR effect. Combining active targeting with a pharmaceutically active ligand and the drug molecules encapsulated within a nanoparticle formulation can improve the EPR effect in a targeted tumor. Bypassing the EPR effect in cases of tumors with low or non-EPR, vascular targeting, or the use of triggerable nanocarriers to release the payload intra-vascularly can be used to enhance dr accumulation in spite of a low or non-EPR effect. To address the heterogeneity in EPR-mediated tumor targeting, direct or indirect imaging techniques, employing either nanotheranostics or companion nano-diagnostics to monitor the biodistribution and tumor accumulation or using standard imaging probes and protocols to visualize tumor blood vessels and the TME are required. Further, EPR-based tumor targeting can help to pre-select a patient for individualized therapies [19,20,21,22].

Thus, complementary active targeting with passive targeting, enhancing circulation, tumor accumulation, drug penetration in the target cell and finally release into the cytoplasm for action through circulation, accumulation, penetration, internalization, and release (CAPIR) cascade to improve the EPR effect is necessary for the development of effective cancer therapy and its translation to the clinic [23].

## 2. Passive Versus Active Tumor Targeting

Active targeting was at first employed to enhance the EPR-based drug delivery as a complementary approach with passively targeted drugs to improve tumor accumulation by nanoparticles to increase targeting efficiency and enhance their retention at targeted tumors [21]. Passively targeted drugs, which are dependent on the EPR effect, may not be sufficient to achieve effective targeting at target sites. However, a meta-analysis of preclinical data indicated that a median of only about 0.7% of injected dose (ID) of nanoparticles actually reaches the target tumor [21]. Several pre-clinical studies have also shown that only 0.1 to 0.2% of the ID are effective against cancer cells and show anticancer therapy with significant patient benefit [20,21].

Active targeting approaches are necessarily much more complex than a passive one. Several challenges associated with these active targeting strategies include physiological barriers and tumor heterogeneity and complex design and engineering needed for these drug delivery systems. The latter may pose major challenges and complicate pharmaceutical development and scale-up under GMP production and, significantly, to the overall cost of the therapy. In spite of several difficulties, one major advantage of active targeting is the ability to target sites disseminated throughout the body, including hematological malignancies and metastatic cancers in which the EPR is not effective [21].

Both passive and active targeting have their own limitations. To ensure clinical success of active targeting, pre-clinical tumor models need to be significantly improved to ensure effectiveness against diseases including solid tumors, hematological malignancies, and metastasis. There are significant barriers to passive targeting resulting in very low tumor accumulation leading to reduced therapeutic efficacy. Passive targeting may not distinguish between normal and diseased tissues. On the other hand, in cases of active targeting, increasing accumulation into tumor cells cannot guarantee the delivery of desired therapeutic agents to the target cells, as drug release may be hindered by the components within the cells. Moreover, endosomal escape of the drug and initiation of drug activating mechanisms is always challenging for targeted delivery. Conjugated nanoparticles may compromise the stealth capacity of the polymer because PEGylating may not be at a sufficient level. Encountering the tumor cell over-expressing receptors proteins without hurdles is a major limitation in targeted delivery. If the stealth properties of the nanoparticles are compromised, then the carriers may be rapidly uptaken/absorbed by the liver, spleen, and other RES organs, resulting in a very low accumulation of drugs in the target tumor.

For both passive and active targeting approaches, the development of companion diagnostic imaging technologies to evaluate the targeting efficiencies is very important. Selection of suitable patients and modifying treatments for specific patients may improve tumor accumulation, efficacy, and therapeutic outcome reducing the adverse effects, unnecessary treatments, and overall health expenses. Finally, active targeting can be used to complement passive targeting for better treatment results.

## 3. Factors Affecting the EPR Effect

The EPR effect has been observed by researchers working in cancer therapeutics for a long time. The preferential accumulation of these nanoparticles in the tumor region is a much more complex aspect than initially envisioned. This process includes several biological processes, including angiogenesis, hemodynamic regulation, vascular permeability, lymphangiogenesis, and heterogeneity of the tumor microenvironment. There is a lot of subject-to-subject variabilities related to these above-mentioned factors. The accumulation of the nanoparticles also depends on various factors, such as the physicochemical properties of each material. A rapidly growing tumor needs an enhanced blood supply as the blood vessels surrounding the tumor are enough to provide the oxygen required for cell growth. New blood vessels are formed to meet the nutritional demands of the tumor cells [24]. The process of angiogenesis surrounding the tumor is rapid, and due to this rapid growth, the blood vessels are irregular with discontinuous epithelium and lack of a basal membrane, constituting a leaky vasculature with fenestrations of 200 to 2000 nm [25,26,27]. This allows enhanced permeation of the blood components as it reduces resistance to extravasation into the tumor interstitium.

Unlike normal tissue, tumors have defective lymphatic drainage resulting in minimal uptake of their interstitial fluid [28]. Molecules smaller than 4 nm can be reabsorbed and diffuse back into the circulation, whereas the diffusion of larger nanoparticles is hindered by their hydrodynamic radii, which results in the accumulation of these nanoparticles in the tumor interstitium [29,30,31].

### 3.1. Extravasation

**Figure 1:**
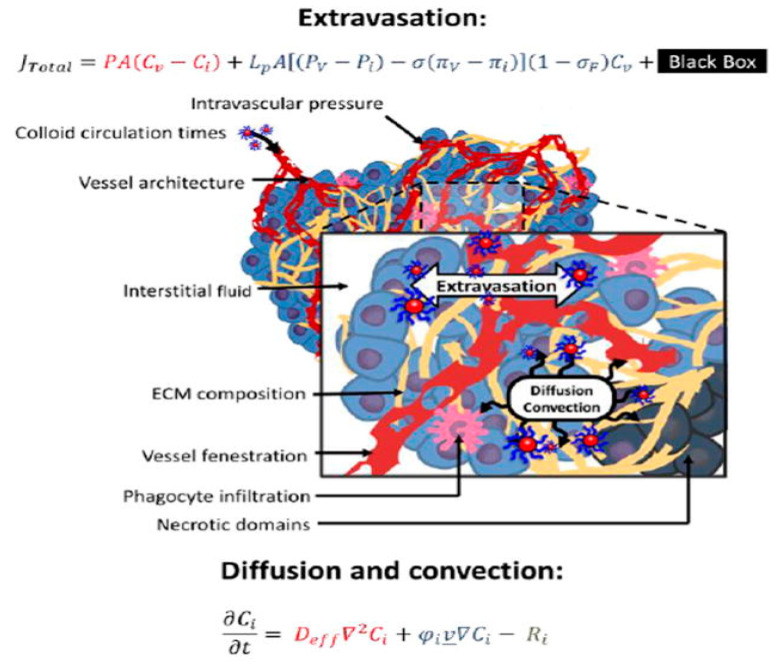
The two phenomena of extravasation and later diffusion and convection of the colloid in the extracellular matrix both result in enhanced permeation and retention of the nanoparticles [32].

The concentration of colloids in the blood regulate extravasation with respect to the permeability of the vascular wall to nanoparticles and the nature of the extravascular environment as shown in Figure 1. The equation below describes the total flux of the material into the tumor, which is an additive function of diffusive and convective forces along with an unknown phenomenon denoted as *Black Box* [32].

*J_Total_* = *PA* (*C_v_* − *C_i_*) + *L_p_A*[(*P_v_* − *P_i_*) − *σ*(*π_v_* − *π_i_*)](1 − *σ_F_*) *C_v_* + *Black Box*

The browninan motion of the blood colloids creates a positive net flux towards the interstitium when a gradient occurs between vascular (*C_v_*) and interstitial concentrations (*C_i_*) [30]. The permeability (*P*) of the wall and the area (*a*) of the wall are measured by a modification of Fick’s law. The diffusion coefficient of the colloid and restriction of passage by the vascular barrier is incorporated in permeability. The physicochemical properties of the colloid and the vessel wall equally affect the hindrance [26].

A convective force is generated due to the discharge of fluids from the vessel. The startling law describes the flux of the fluid, and the filtration coefficient of the fluid through the vessel is denoted by *L_p_*. The hydrostatic pressures of vascular and interstitial parts are denoted by *P_v_* and *P_i_*. Vascular and interstitial oncotic pressures are denoted by *π_v_* and *π_i_*, respectively [30]. The *σ* is a capillary osmotic reflexion coefficient, which reflects the permeability of the capillary to large molecules such as proteins. It also describes how effective it is at pulling back fluid into the vascular space due to the oncotic pressure gradient. *σ_F_* and *C_v_* are the drag of the colloid by the fluid and colloid concentration in the vascular compartment, respectively.

The black box in the equation denotes the unknown phenomena by which colloids extravasate and reach the tumor. This lays the path to further exploration of the EPR effect. Some researchers believe that interactions with endothelial cells could cause increased permeability of the vessel. For example, cationic charges on the nanoparticles can cause more interactions and thus more permeability. Others consider these interactions a part of absorption and endocytosis by the endothelium [33,34,35,36]. Another important factor to consider for the black box is uncertain as a predictor of the concentration in the vasculature available for extravasation. The presence of phagocytic cells can cause an increase in the concentration of the nanoparticles in the vasculature of the tumor microenvironment due to the characteristic interaction of the nanoparticles to interact with phagocytic cells. [37]. Furthermore, the payload of these nanoparticles might have different properties compared to the nanoparticles. Thus, their release kinetics and their interactions within the tumor also have to be accounted for.

### 3.2. Diffusion and Convection in the Interstitium

The movement of the colloids once extravasated into interstitial fluid containing cancer, stromal cells, and extracellular matrix are guided by diffusive and convective forces. This is further described in the equation below:

$$\frac{\partial C_{i}}{\partial_{t}}=D_{eff}\nabla^{2}C_{i}+\varphi_{i}\underline{\nu }\nabla C_{i}-R_{i}$$

The change of the interstitial concentration over time results due to the diffusive component and convective component along with the effects of the tumor microenvironment on the colloid transport (*R_i_*).

### 3.3. Tumor Vasculature and Biology

Untamed growth of the cells and angiogenic factors contribute to the disorganized vasculature and congested extravascular environments. These structural imperfections can promote the EPR effect and accumulation of nanoparticles in the tumor. The new blood vessels being formed are disordered and discontinuous with many fenestrations [38]. The cancer cells dictate the blood vessel architecture by releasing angiogenic factors [36]. Hence the type of cancer dictates the degree of leakiness of the endothelium and enhanced vascular permeability to macromolecules. They also depend on what stage the cancer is and the site it is located at [26,27,39]. These irregularities in the architecture of the vessels affect the flow and the pressure in the blood vessels, which can dictate the permeation and retention of the colloids. A highly proliferative tumor mass can also exert pressure on the blood vessels to hinder their perfusion. Thus, reduced pressure can lead to decreased convective forces and increased extravasation of both blood components and nanoparticles [26,28,38].

### 3.4. Tumor Extravascular Environment

The tumor extravascular environment is a haphazard, crowded entanglement of collagen fibers and glycosamine glycans (GAGs). Unlike normal tissues, the tumor microenvironment has solutes, proteins, and debris distributed unevenly [30,40]. Interstitial hydrodynamic and oncotic pressures play a key role in the convection of nanoparticles through the vascular wall, which are directly affected by the haphazard traffic of fluids [41]. The extracellular matrix will regulate the diffusive and convective forces that regulate the movement of nanoparticles once extravasated. The diffusive coefficient in the tumor interstitium is lower than in simple solutions for colloids and several in vivo and ex vivo studies have shown the same [42,43]. The viscosity of the environment and the diffusive paths can be altered by GAGs covalently linked to proteins such as collagen. The colloids of different sizes show high and low mobilities due to GAG chains that are organized in low and high viscosities, essentially making it a two-phase transfer process [43].

Resistance exerted on the interstitial transport correlated to the content and degree of organization of collagen in the ECM. The use of the collagenase enzyme may break the protein entanglement and restore mobility and help diffusion. Some research groups have shown that intratumoral injections of collagenase can enhance the mobility of viral vectors of 150 nm in size [43,44,45].

On the other hand, GAG-disrupting enzymes have not shown any significant effects. There were instances where injecting hyaluronidases decreased the diffusion of macromolecules and injecting heparinases that cleave heparin sulfate moieties restored the mobility of cationic macromolecules. The latter might be due to a decrease in the absorptive interactions of the colloids in the ECM [42,43,44].

Cells of the mononuclear phagocytic system have the tendency to extravasate to the tumor interstitium and inhibit the movement of the nanoparticles toward cancer cells. This happens due to the affinity of these macrophages to the colloids resulting in increased phagocytic activity [46]. Zamboni et al. showed that increased liposomal accumulation was seen in xenografts of ovarian cancer with increased CD11 positive cells in comparison to melanoma cells with lower dendritic cell expression [37]. The age of the individual also affects the clearance of nanoparticles such as liposomes. Older patients or patients with hepatic metastases have been shown to have higher exposure levels. Furthermore, older patients also tend to have lower hematological toxicities compared to patients below 60 years of age. This suggests that mononuclear phagocytes interact with nanoparticles, which could then affect the pharmacodynamics of the nanoparticles [47,48].

### 3.5. Changing Tumor Biology to Improve EPR

The tumor microenvironment can be optimized to enhance the distribution of nanoparticles in a tumor. As mentioned earlier, intratumoral injection of the enzymes to reorganize the extracellular matrix is an effective method. Similarly, reshaping the perivascular environment has been utilized in photo-immunotherapy or to deliver low molecular weight drugs. Preclinical models have shown increased accumulation and retention of the nanoparticles and oncolytic viruses with these approaches [45,49,50]. Increasing the perfusing pressure via improving transvascular convective movement is another approach. Administration of hypertensive drugs such as angiotensin II resulted in increased extravasation of the colloids with sufficient affinity to bind to the tumor and avoid being translocated back into the circulation. Moreover, the administration of angiotensin converting enzyme (ACE) inhibitors such as enalapril resulted in an increased accumulation of antibodies [2]. The ACE inhibitor blocked the degradation of bradykinin (a potent physiological vasodilatory peptide), which increased the permeability to large molecules [41,51]. Administration of both the ACE inhibitor and angiotensin II is a good approach to increase the EPR as angiotensin II will counteract the hypotensive effect of ACE inhibitor enalapril. EPR can be increased by employing other vasodilatory agents using nitric oxide, prostaglandins, and carbon monoxide [52,53,54,55,56].

### 3.6. Physicochemical Factors That Affect EPR

The physicochemical properties of the colloids play a key role in the EPR effect by altering their extravasation. The physicochemical properties of any external material for therapeutic or diagnostic purposes are important in drug delivery as they impact the way the host defense mechanisms clear them from the systemic circulation [57,58]. The size, charge, and shape of the nanoparticles are the important physicochemical properties that dictate the EPR effect. Table 1 lists the characteristic size, charge, and shape needed for increased enhanced permeation and retention.

Furthermore, total blood exposure of these nanoparticles should be the key factor influencing the accumulation of the nanoparticles inside the tumor. The concentration of these colloids influences the diffusive and convective forces necessary in controlling the amount of extravasation into the interstitium. The efflux of the nanoparticles from the tumor can be hindered by maintaining higher concentrations in the bloodstream [32]. The above physicochemical properties can help the drug delivery scientists to design the nanoparticle in such a way as to increase the EPR effect and increase the drug concentration at the site of action.

## 4. Heterogeneity of EPR: A Clinically Relevant Phenomenon

In the last couple of years, research reports citing nanocarriers and EPR effect-based therapies have been increased markedly. The basic rationale for tumor targeting via EPR effect has been presented in thousands of research papers that claim improved therapeutic potentials and consider this phenomenon a royal gateway. However, at present, scientists and oncology specialists are of a view that these therapies are failing in the clinic and that the EPR effect is misinterpreted and overrated. This approach, “one size fits all,” worked in lab animal tumor models but not in humans, possibly because they were transient in nature, thus limiting the bench to bedside translation of most targeted tumor therapies. The heterogenous outcomes of clinical trials have led to a new understanding that the EPR effect varies greatly between lab animals and humans as well as among different tumor types and metastases within the same individual. To address the complex nature of the EPR effect, the research is now moving towards a custom-fit approach to personalize the patient therapy for better outcomes and to identify the most responsive patients from clinical trials [20,65,66,67,68].

Human tumors differ greatly from animal tumors with respect to the rate of growth, size of the tumor, tumor-to-body weight ratio, and heterogeneity of the tumor microenvironment that collectively alters the pharmacokinetics of most drugs. The degree of tumor heterogeneity varies in different types of tumors as well as with the same types of tumors in different patients. Thus, complete control and performance monitoring throughout therapy might help develop successful clinical trials [69,70].

### 4.1. Heterogeneity of Tumor Blood Flow and Hypoxic Areas

The abnormal tumor growth requirements for nutrients and oxygen direct the neighboring tissues to proliferate and lead to the ingrowth of a vascular supply. The imbalance between oxygen supply and demand leads to hypoxia. The irregular branching order with enlarged vessels and chaotic blood flow within different parts of a tumor lead to the heterogenous distribution of drugs. The resulting hypoxic parts of tumors alter the EPR effect by activation of fibrinolysis, clotting, or bleeding in some tumor parts, result in poor delivery of drugs [65,71]. The activation of hypoxia-inducible factor (HIF) signaling is regulated by hypoxia through multiple mechanisms, including overexpression of Jagged 2 and Notch signaling, activation of CD24 expression, induction of integrin-like kinase, ILK and elevated levels of hypoxia induced genes such as B lymphocyte-induced maturation protein-1 (BLIMP1) that collectively promote metastatic stem cell phenotypes [72,73,74,75,76,77].

### 4.2. Heterogenous Vascular Permeability and Extravasation

The rapid tumor growth and improper development of blood vessels in murine tumors result in a much leakier vasculature as compared to human tumors that can lead to misinterpretation of the EPR effect [78]. This is generally not the case in humans, where not all tumors manifest a leaky vasculature and resultant enhanced permeability to macromolecules. However, there are certain human tumors that are very leaky, well-vascularized, and overexpress VEGF. Despite the above notion, some human tumors respond nearly as hypothesized, and the discovery of U.S. FDA approval of Doxil for AIDS-related Kaposi’s sarcoma can be noted here. The liposomal doxorubicin extravasated and accumulated intact in tumors with a leaky vasculature [79].

### 4.3. Heterogenous Penetration

The clinical isolates from a variety of cancer patients support tumor related abnormal blood coagulation. This process starts when tumor cells erode the neighboring normal or tumor blood vessels resulting in microscopic hemorrhages. The insoluble fibrin (IF) clot formation and replacement with collagenous tissues start immediately to compensate for the tissue damage. This silent process called ‘malignant cycle of blood coagulation’ in cancer patients is similar to the normal wound healing process, however these fibrin clots survive with cancer cells. This fibrotic stroma provides a barrier to the penetration of chemotherapeutics and resultant treatment failure. This stromal barrier is more prominent in solid cancers that are invasive and hypercoagulable such as glioblastomas, pancreatic cancers, and stomach cancers [10,11,12].

When moving from the periphery to the center of tumors, drugs face heterogenous vessel stress and collapse due to proliferating cells as the density of tumor and interstitial fluid pressure increase that further hinders the transport of drug molecules [80]. The human tumors differ with respect to pericyte coverage (smooth muscle actin cells attached to endothelium) in that it is high and compact while low and loose in murine models. The tumors with a poor prognosis (brain tumors, renal carcinomas) have a fibrotic interstitium with more pericyte coverage (60–70%) as compared to tumors with better prognosis (ovarian carcinoma, colon cancers) with about a 10% coverage [81,82].

Adequate vascular pericyte coverage is crucial in normal cells’ maintenance of the blood–brain barrier (BBB), where the loss of these cells leads to various brain disorders. The neoplastic pericytes derived after genetic modifications of glioma stem cells (GSC) develop a blood tumor barrier (BTB) that hampers the penetration of chemotherapeutics and results in treatment failure. The preferential overexpression of BMX-kinase in GSC-derived pericytes makes it a suitable target to disruption of the BTB and enhance the penetration of anticancer drugs. Zhou et al. identified ibrutinib as a potent tumor pericyte-disrupting drug. Their findings suggest that adequate synergism can be established by combining this treatment with some poorly penetrating anticancer drugs [83]. The pericytes serve as a gatekeeper against tumor progression and metastasis to other organs of the body. The clinical data also suggest that low pericyte coverage results in high mortality of cancer patients [84]. The basement membrane (BM) and extracellular matrix (ECM) also play an important role in determining the porosity and stiffness of human tumors that lead to heterogenous penetration, poor prognosis, and treatment failure [66]. Lee et al. reported non-invasive and cost-effective pulsed high intensity focused ultrasound technology as an ECM remodeling strategy as an alternative to intratumoral injection of collagenase and hyaluronidase for deep penetration of nanoparticles [85].

## 5. Strategies to Overcome Heterogeneity

Various treatment modalities based upon specific pathophysiology of tumor and EPR effect have been proposed with more than 7350 citations over the first report of EPR (as of June 2021 from Google Scholar). The CAST therapy received considerable attention from researchers after the successful development of new strategies to achieve highly localized concentration of topoisomerase-1 inhibitor, SN-38, conjugated with monoclonal antibody (mAb) targeted against collagen-4. This newly developed immunoconjugate was optimized to bound with stromal collagen creating a scaffold with sustained release of anticancer agent [13,14]. Gebleux and coworkers proposed non-internalizing antibody drug conjugates, ADC, that rely on extracellular release of drug thus preventing antigen barriers [15]. ADC might overcome heterogeneity of tumors by utilizing TME to facilitate cleavage of linkers and payload release [16]. Tumor endothelial marker-8 (TEM-8) is overexpressed in perivascular stromal cells and can be used as a useful stromal target for locally triggered drug release from anti TEM-8 ADC [17]. The heterogenous antigen distribution in malignant cells and the difference in targeted gene copy number among patients are serious challenges for researchers, and a single mAb may not be effective for all patients [14].

Many approaches have been proposed for mAb-based tumor targeting and mechanisms to overcome therapeutic resistance that is caused by the heterogeneity of tumor antigen and also the resistance executed by TME, including inefficient delivery to the tumor, alteration of effector functions in the TME, and Fc-γ receptor expression diversity and polymorphism. mAbs-based therapies are potential approaches to overcome these barriers using several diagnostic and prognostic biomarkers for envisaging response to mAb-based therapies [18].

EPR-effect has been proved by many preclinical animal models. However, results obtained from animal models are usually conflicting with clinical observations. Unlike hematological malignancies, in solid tumors, administered anticancer agents (ACA) must diffuse through the tumor mass, overcoming cancer stromal barriers and tumor mass itself. It has been demonstrated that hypercoagulability caused by cancer stroma, and the more aggressive cancer, the greater the deposition of insoluble fibrin (IF) in cancer tissue [14]. An ant-IF mAb was developed and conjugated with an ACA using V-L-K linker. The resultant ADC drug linker is degradable by plasmin. The plasmin is activated during the IF formation only. ACA is released from the ADC drug particularly when the conjugate binds to the IF. This novel approach was beneficial to deliver ACA to tumor cells through the stromal barrier due to the small size of the drug [14].

Numerous strategies have been used to modify the abnormal tumor microenvironment in humans by combination with nanomedicines. The direct permeability enhancement by various methods has been explored that take advantage of the EPR effect and facilitate the delivery of drugs/macromolecules inside tumors. Examples include the selective inhibition of angiotensin-converting enzymes [86,87], generation of NO or CO within tumors [56,87,88], blockage of VEGF and other angiogenic signaling factors [89,90,91], inhibition of pericyte recruitment and BM activation [92,93], and image guiding systems [94].

The recent advancements and technological innovations have allowed novel insights into the drastic differences between murine and human cancers that can hamper the clinical translation of tumor-targeted nanotherapeutics. The laboratory-established models are not true representatives of human cancer in many respects and require modifications to explain the heterogenous events responsible for compromised EPR effects in humans. To maximize the clinical outcomes of investigational cancer therapeutics, new strategies to mimic the individual tumors are required that closely recapitulate the patients’ responses to preclinical drug testing [95,96]. This approach provides the potential for guided clinical decision-making in translational cancer research by individual performance metric calculation. Tailoring the cancer therapy to patient groups that are more prone to respond and benefit from the investigational treatment offer a potential solution to overcome the heterogeneity of the EPR effect. Patient-derived tumor xenografts (PDX) involve the engraftment of specific tumor tissues in immunocompromised mice. Izumchenko et al. integrated PDX models via implantation of 92 different solid cancers from a 237 cohort of patients into immunodeficient mice. They analyzed and compared the patient responses and PDX models after whole exome sequencing. Their findings suggested that these models accurately replicated the patient outcomes over a repetitive course of therapy, enabling an oncologist to assess the patient-specific cellular events [97]. The mouse models offered numerous benefits, such as their small size, ease of reproduction, transgenicity, and closely mimicked physiology. However, various limitations involving mice such as high cost, complex genetic manipulations, and prolonged duration of experimentation have forced researchers to utilize alternatives. Numerous current publications reported the use of chick chorioallantoic membrane (CAM) and Zebrafish for implantation as alternatives to mice. Hu and coworkers demonstrated that CAM is an efficient system to analyze pilot drug responses in patients with bladder tumors, accelerating the discovery of critical molecular mechanisms [98]. Mercatali et al. studied the metastatic potential of breast cancer after injecting primary culture of bone metastasis derived from a 67-year-old patient into zebrafish embryos. Their findings suggested zebrafish are a suitable substitute for mouse models and provide for a better understanding of chemotherapeutic sensitivity and prognostic marker identification [99]. Table 2 shows the status of some patient-derived tumor models.

## 6. Targeting Tumor Tissues via an EPR Effect

**Figure 2:**
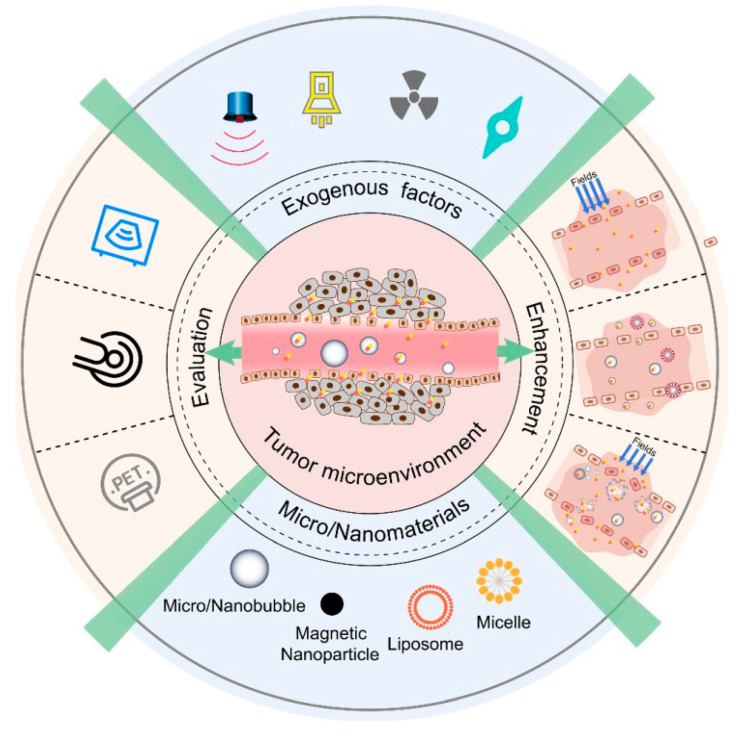
Common strategies utilizing the EPR effect [109].

Since the discovery of the EPR concept, it has been utilized widely for many applications (Figure 2), especially for the delivery of anticancer drugs. The EPR effect helps promote a favorable biodistribution of nanoparticles in blood and a high level of nanoparticle accumulation in solid tumors. However, for the optimal development of nanoparticles for enhanced drug delivery by EPR effect, multiple factors should be considered, including blood half-life of nanoparticles, minimal nonspecific delivery, and effective elimination from the body [108].

The EPR effect discovery was a milestone in drug delivery systems, and expectations for utilizing this effect in a selective anticancer drug delivery were high. However, the transition of nano-drug delivery medicine from benchtop to clinic has been very difficult. An EPR effect-mediated drug accumulation has been proved with various natural and synthetic molecules with molecular sizes greater than 40 kDa or 7 to 8 nm in diameter. Encapsulation of small molecules inside macromolecular vehicles, including liposomes, nanospheres, or polymeric micelles, led to full utilization of the EPR effect and made it a universal method for targeting the tumor side known as passive targeting. The characteristics of the EPR effect are at disposal for this method of targeting, including (i) defective architecture of blood vessels, known as a “leaky vasculature,” with large gaps (around 400 nm) between capillary endothelial cell linings; (ii) overproduction of vascular mediators including bradykinin and nitric oxide [NO]); and (iii) improved retention of the macromolecules in tumor tissue due to impaired lymphatic recovery [110,111].

### 6.1. Chemotherapeutics Targeted through EPR Effect

Taken together, it can be said that successful drug delivery via EPR effect is based on the properties of the molecule/carrier, including (i) biocompatibility; (ii) molecular size greater than renal clearance threshold (40 kDa); (iii) neutral or slightly negative charge; (iv) drug retention time greater than a few hours (v) circulation time longer than a few hours [65,110]. Among these factors influencing the EPR effect, the most important are molecular size and biocompatibility. To prevent renal clearance, low molecular weight drugs are conjugated to polymers or to natural blood components that circulate long in the plasma, such as serum albumin or lipoproteins. The best-known example of such a polymer is poly (styrene-maleic anhydride) (SMA). It has been proven that the conjugation of peptides and proteins with a polymer of this type (1.5 kDa) allows extending the circulation time of anti-cancer proteins and peptides by binding the conjugates to plasma albumin. It has also been shown that conjugation with SMA protects proteins from enzymatic degradation and reduces the immunogenicity of modified proteins. An example of such a conjugate is neocarzinostatin and SMA (SMANCs) used for the treatment of hepatoma, which accumulates in solid tumors through the EPR effect and which was the basis for other conjugates based on the same mechanism of action [112,113,114]

The copolymer to which a wide variety of anti-cancer drugs including doxorubicin, has been attached is based on *N*-(2-hydroxypropyl)methacrylamide (HPMA) for delivery to tumors via the EPR effect [115,116,117]. Conjugates of HPMA copolymer with an anticancer drug are active in many models and have low immunogenicity, which makes it possible to improve such a conjugate to target and control its subcellular localization of the drug based on its mechanism of action [116,118,119].

Another method for drug delivery through the EPR effect is to entrap the drugs into nanoparticles. The disadvantage of this solution is that large-sized nanoparticles will be absorbed by organs of the reticuloendothelial system (RES), such as the liver and spleen, resulting in slower elimination from the body and a potential for toxicity [120]. For this purpose, the concept of stealth liposome was developed. Conjugation of phospholipids with polyethylene glycol (PEG) leads to the formation of a protective, hydrophilic layer on the liposome surface. This layer prevents recognition of the liposomes by opsonin and other complement components, thereby preventing clearance through the RES system and increasing the half-life of the liposomes. As stealth liposomes, the appropriate size of the liposomes prevents loss due to renal filtration, while pegylation, in turn, ensures that the RES system does not recognize the nanoparticles, which leads to the preferential accumulation of liposomes in tumor tissues through the EPR effect [121,122]. The first liposomal formulation that met these guidelines and was approved by the FDA was Doxil^®^, a formulation containing doxorubicin hydrochloride used in the treatment of AIDS-related Kaposi’s sarcoma, multiple myeloma, and ovarian cancer using the EPR effect. To date, the FDA has approved many other liposomal formulations such as DaunoXome^®^, which contains daunorubicin, and Marquibo^®^, which contains vincristine sulfate for cancer therapy [123] as well as other types of nanoparticle including polymeric micelles containing paclitaxel (Genexol^®^ PM), micelles built with PEG and a poly(γ-benzyl L-glutamate, containing cisplatin [124] and albumin nanoparticles with paclitaxel (Abraxane^®^) [125]. A new strategy to overcome the dilemma between the EPR effect and renal clearance was the development of multifunctional particles such as FeTNPs. These molecules were designed based on the coordinated interaction of phenolic groups and iron, composed of ferric iron, tannic acid (TA), and poly (glutamic acid) -graft-methoxy poly (ethylene glycol) (PLG-g-mPEG). FeTNPs are characterized by their effective accumulation in tumor tissue based on the EPR effect and the possibility of being disassembled dynamically by deferoxamine mesylate (DFO) to accelerate the elimination of nanoparticles, thus reducing the potential for toxicity [126].

Another extremely important physicochemical parameter that affects the time of systemic circulation and intratumoral processes is the presence of a surface charge, which can control the opsonization profile of the material, its recognition by the MPS cell, and its overall plasma circulation profile. The desired parameter has a neutral or slightly negative surface charge, while a positive charge was believed to lower the circulation time. However, the non-pegylated, positively charged liposomes containing 1,2-diacyl-trimethylammonium propane (DOTAP) lipid have been shown to have higher tumor-to-surrounding tissue ratios than their negative or neutral counterparts. The positive charges are believed to promote NP interactions with tumor blood vessels and compromise their predisposition to deeper diffusion in the tumor while preventing their redistribution in the systemic circulation. This phenomenon has been exploited for therapeutic purposes by targeting an endothelial tumor vessel with anti-angiogenic and antitumor drugs in preclinical and clinical models [33,127].

### 6.2. Targeting DNA, siRNA, and Other Nucleotides

The main strategy to target nucleic acids via the EPR effect is to encapsulate/conjugate them with nanoparticles. Similar to anticancer drugs, for specific tumor delivery of siRNA, sufficient longevity of loaded siRNA carriers is required [128]. Currently, polyethyleneimine (PEI) is one of the most studied and successful cationic polymers for nucleic acid delivery, including siRNA. Unfortunately, PEI of high molecular weight did not show high transfection efficiency and also showed significant systemic toxicity. To reduce the toxicity of a PEI-based delivery system, polyethyleneimine is most often combined with other polymers such as PEG [129] or dendrimers [130]. These have led to the successful clinical use of PEI to deliver sensitive genetic biomaterials [129].

To improve the PEI-based genetic material delivery system, a conjugate of a lipid, PEI, and polyethylene glycol (PEG) with a hypoxia-sensitive linker (azobenzene; a derivative of nitroimidazole) was developed. The advantage of this approach is that under hypoxic conditions, the azobenzene linker is degraded, thus releasing the protective PEG layer, exposing the siRNA to allow hypoxia-dependent cellular uptake. This method uses the enhancement of the EPR effect by targeting the carrier to a specific tumor environment. This strategy may allow for an effective supply of genetic material and can be used as a therapy for drug-resistant tumors [131,132].

### 6.3. Targeting Imaging Agents

The EPR effect is an important tool for specific nanoparticle targeting in cancer therapy as well as for diagnostic purposes. Diagnostic techniques such as fluorescence imaging, positron emission tomography (PET), magnetic resonance imaging (MRI), computed tomography (CT), or single-photon emission computed tomography (SPECT) require delivery of small molecules to a tumor site, which is a challenging task [133,134]. Nowadays, imaging plays an important role in clinical oncology by serving as the main tool to identify solid tumors and determine therapeutic responses. Unfortunately, imaging techniques including CT and MRI have limited sensitivity and thus, cannot provide specific and functional information on the disease due to usage of non-targeted contrast agents. Therefore, there is a definite need for new contrast agents or modified existing ones. Significant progress was seen with recently developed biodegradable nanostructures of iron oxide for MRI and luminescent quantum dots (QDs), a new class of light-emitting particles. The nanoparticles were built of PLGA-mPEG polymer and showed prolonged circulation half-life and improved imaging effects [135,136].

Recently, liposomes containing 89Zr have also been formulated for photodynamic therapy and PET imaging. Liposomes with a multicompartment-membrane were developed containing tween-80, where 89Zr was conjugated with a deferoxamine chelator with tetrakis (4-carboxyphenyl) porphyrin. These radiolabeled liposomes showed enhanced EPR effects, improved photodynamics, and in vivo stability [137]. Copper-64 containing PEGylated liposomes were also developed to clearly observe the EPR effect through PET imaging [138].

Similarly, for fluorescent imaging, various strategies have been developed. Fluorescent dyes have been conjugated to macromolecules to enhance the EPR effect, including tetramethylrhodamine isothiocyanate (TRITC)-conjugated, with high-molecular-weight [MW 67,000] bovine serum albumin (BSA). Another polymer, *N*-(2-hydroxypropyl)methacrylamide (HPMA) (13 kDa), was also conjugated with zinc protoporphyrin (ZnPP). Formed micelles were about 80 nm in diameter and produced a clear tumor image similar to BSA-TRIC conjugate [139].

Nowadays, superparamagnetic iron oxide nanoparticles (SPION) are being used as an MRI contrast agent for tumor imaging. SPION are usually built of magnetite (Fe_3_O_4_) or maghemite (Fe_2_O_3_), encapsulated within an aqueous core. The marketed SPION contrast agent that has been used for tumor imaging is known as Endorem^®^. Modification of SPION with the ultrasmall size is USPION, known as Sinerem^®^. These nanoparticles with a size below 50 nm are mostly used for the detection of brain tumors [140].

Although we understand more about the EPR effect and many approaches have been taken to utilize this phenomenon in cancer treatment and diagnosis, there are still many challenges left. Thus, significant research has been focused on the enhancement of the EPR effect.

## 7. Approaches for Promoting EPR of Nanodrugs in Cancer

There is a large intra- and inter-personal heterogeneity in EPR-based tumor targeting, which is reflected in the outcome of clinical trials showing unexpected lower success rates. Based on the nature, heterogeneity, and complexity of the EPR effect, the development of systems and approaches for enhancing, combining, bypassing, and imaging the EPR tumor-targeting are crucial.

In healthy tissues, low MW drugs easily extravasate out of blood vessels, whereas nanodrugs are often unable to do so because of size. On the other hand, in tumors, abnormally wide fenestrations in the blood vessels allow the extravasation of nanoparticles with sizes of up to several hundreds of nm. Additionally, the relative absence of lymphatic drainage leads to an effective and selective accumulation of nanodrugs in tumors.

Multiple vascular and TME parameters contribute to heterogeneity in EPR-mediated tumor accumulation. Biological barriers that contribute to heterogeneity in EPR-based tumor targeting include high cellularity, dense ECM, hypoxia, interstitial fluid pressure (IFP), vascular permeability, endothelial cell receptor expression, and vascular maturation at the vessel level.

Many pharmaceutical and physical approaches can be used to enhance tumor accumulation and efficiency of the EPR-based drug delivery. Important pharmacological approaches include modulating VEGF signaling with angiotensin agonists and antagonists, TNFα, vessel-promoting treatments, and nitric oxide-producing agents. Physical approaches include hyperthermia, ultrasound, radiotherapy.

Regulation of particle size and encapsulation in micelles can improve circulation time and enhance accumulation via the EPR effect. Encapsulation of doxorubicin in liposomes, Doxil, enhances plasma half-life by up to 2–3 days compared to free drug. In many liposomal and micellar nanodrug formulations, surface modification with the stealthy polymer, PEG, decreased aggregation, opsonization with plasma protein and enhanced the circulation half-life [20].

Anti-angiogenic drugs can be used to deprive tumors of nutrients and oxygen. For instance, at an intermediate dose of anti-angiogenic drug, bevacizumab was employed to normalize the disorganized tumor vasculature into a highly vascularized one to enhance drug delivery. The use of intermediate doses of Doxil or Abraxane enhanced the accumulation of paclitaxel in tumors by restoring convective drug delivery with reduced IFP, and without decreasing the concentration of doxorubicin in a size-dependent manner. Bevacizumab-mediated vascular normalization enhances the antitumor response of Doxil-based chemotherapy of ovarian cancer in a clinical setting [141].

TNFα is a potential inflammatory molecule. It led to a 10-fold higher EPR-induced accumulation of radio-labeled liposomes in mouse tumors compared to non-TNFα treated ones. Fibromuna, an antibody fused to TNFα used in melanoma treatment, has been used in combination with doxorubicin for sarcoma treatment and in combination with melphalan used for isolated limb perfusion to avoid amputation of a cancerous limb [142,143]. Although clinical trials with several TNFα-based drugs are promising, their use has been limited to local delivery due to their systemic toxicity [20].

Accumulation by EPR in tumors can be enhanced with Angiotensin II receptor blockers (ARB) that promote vessel permeability and dilation through the loosening of the endothelial cadherin-mediated intracellular connections [53]. The ARBs can modulate the expression of ECM, leading to vessel decompression and an enhanced EPR [144]. For example, losartan can decompress tumor blood vessels, increase vascular perfusion and EPR-targeted drug delivery [145]. Instead of increasing vessel permeability and dilation of vessels using ARBs, vasoconstriction using angiotensin II can also be used to enhance the EPR-mediated drug delivery. Angiotensin II can induce systemic vasoconstriction in healthy blood vessels. However, since tumor blood vessels are immature and lack a uniformly differentiated and structured smooth muscle cell layer, they do not contract in response to AT-II. As a result, the AT-II drugs can enhance the EPR drug accumulation through an increased blood flow into the tumor blood vessels compared to healthy blood vessels. Thus, AT-II can lead to better perfusion of tumor tissues and enhanced EPR-based drug accumulation in tumors. However, this treatment option may be limited to patients with normal BP levels.

Approaches to vessel promotion of increased angiogenesis instead of inhibiting the angiogenesis can result in more vessels and a higher delivery of drugs. Cligentide can bind to αvβ3 integrins to prompt anti-angiogenesis [146]. Verapamil, a calcium channel blocker, induced higher blood flow and enhanced blood vessel perfusion. The combination therapy using cligentide, verapamil, and gemcitabine enhanced survival in a mouse model of pancreatic cancer due to a reduced tumor burden. This combination therapy significantly increased vessel density and reduced hypoxia, exhibiting possible benefits of vessel promotion in combination with chemotherapy. Similarly, erythropoietin in NSCLC models, increased vessel density by 50%, doubled the relative tissue blood volume, through the vessel promotion, which resulted in up to a 100% increased cisplatin delivery [147].

Radiation therapy can increase vascular leakiness through upregulation of fibroblast growth factor [148]. The combination of nanodrug therapy and radiotherapy can enhance the EPR-based drug delivery. Combination therapy using Doxil with radiotherapy in osteosarcoma xenograft mice delayed tumor growth compared to the control [9]. However, un-encapsulated nanodrugs, when combined with radiotherapy, may have severe side effects [149]. In fact, ionizing radiation has an effect on different cell types in TME. Besides increasing vascular leakiness, radiotherapy can prompt MDR and metastasis [150]. Thus, applying radiotherapy in combination with nanodrug therapy required precautionary steps.

Hyperthermia can be used in combination with chemo- and radiotherapy for locally well-defined solid tumors. This can be applied through radiofrequency, microwave-focused ultrasound, and intracavity perfusion. Hyperthermia increases tumor blood flow and enhances vascular permeability and promotes drug and oxygen supply to tumors. Thus, hyperthermia can be used to enhance EPR-mediated drug delivery, especially in non-leaky tumors with a very low level of baseline nanodrug accumulation [151]. Extravasation of 100 nm liposomes was enhanced significantly upon increasing temperature [152]. The combination of hyperthermia with temperature-sensitive nanodrugs can be an effective EPR-based drug delivery system.

In low EPR tumors upon sonoporation, liposome accumulation was enhanced up to 100% [20]. Sonoporation in combination with gemcitabine-based therapy in pancreatic cancer demonstrated positive impact [153]. Usually, CNS drug therapies are not effective due to the BBB. However, sonoporation prompted a spatially and temporally controlled BBB opening facilitating enhanced drug delivery [154]. Ultrasound (US)-mediated brain vascular opening demonstrated that MRI-focused US treatment in combination with Doxil enhanced drug accumulation in a gliosarcoma model [155]. Clinical trials to evaluate minimal US required for safely open the BBB are currently ongoing [156].

Photodynamic therapy (PDT), based on a photosensitizing agent and ROS formation, can damage nucleic acids and proteins, leading to cell death. A mAb-photosensitizer conjugate, panitumumab-IR700, against the EGFR in combination with laser light and liposomal daunorubicin was used to treat tumors [157]. Treatment of tumors with EGFR targeted photosensitizer, prior to daunorubicin treatment led to super-enhanced permeability and retention (SUPR), enhanced tumor accumulation, and therapeutic efficacy. However, clinical limitations of PDT, including penetration depth of laser light and short migration distance by ROS, render the treatment options for wide-spread tumors and metastasis located deep in the body almost impossible.

The pretreatment with intralipid increased accumulation of nanomedicine in tumors due to induced reduction in liver uptake. This increased accumulation, therefore, led to significantly improved therapeutic effects, which were validated by using the Doxil drug. As a fascinating result, intralipid pretreatment prolonged the plasma half-life of nanomedicines in normal healthy mice but not in tumor-bearing mice, which suggests that tumors may be an alternative route of nanodrug delivery when liver delivery is suppressed [158].

The combination of two different drugs within a formulation can be beneficial for EPR targeting. CPX-1 is a liposome containing irinotecan and fluoxuridine in a 1:1 molar ratio enhanced EPR-accumulation of CPX-1 in colorectal cancer [159]. Combination of Abraxane with gemcitabine for the treatment of metastatic PDAC, and a combination of abraxane with carboplatin for the treatment of NSCLC have shown promise in the clinic [160]. Trastuzumab, together with different chemotherapeutics, are used for the treatment of HER2-positive metastatic breast cancer [161]. An EGFR-targeted nanobody linked to a polymeric micelle (PM), nanobody modified DOX-PM, inhibited tumor growth and prolonged survival [162].

The combination of an anti-PD-L1 with an immunogenic cell death-inducing nanoscale coordination polymer (NCP), loaded with oxaliplatin, as well as photosensitizer for PDT showed success in cancer therapy [157]. Combination therapy using nanoformulations suitable for combined PDT and immune checkpoint blockade was also successful against TNBC [163].

Combination of several drugs in a nanocarrier and application ADCs accumulated drugs through an EPR effect and enhanced delivery of all drugs with different mechanisms to the tumor cell, without cross-resistance. This combination therapeutic approach enhances the impact of individual drug components, aids synergistic effects, reduces side effects due to the encapsulation of drugs into a nanocarrier resulting in an enhanced nano-immunotherapeutic outcome [164].

Patients with a low EPR tumor with a non-leaky vasculature need either active delivery or a bypassing of the EPR effect to trigger drug release into the tumor. Several approaches have been developed to deliver nanoparticles without reliance on an EPR effect [5]. Fluorescent peptides that form nanoparticles in situ in tumors, and treatment responses that can be detected by imaging, have been developed [165]. Similarly, an assembly of GNPs and fluorescent contrast agents has been used for the detection of drug delivery in tumors [166].

For low EPR tumors, an injectable nanoparticle generator (iNPG) has been developed to enhance drug delivery to tumors. The iNPG releases the drug-polymer conjugate due to natural tropism and enhanced vascular dynamics and forms self-assembled nanoparticles in situ, which are transported to the perinuclear region to bypass the drug efflux pump. The iNPG system was effective in TNBC, MDA-MB-231, and 4T1 mouse models of metastasis [21,167].

Another EPR-independent approach to improve tumor targeting in low EPR tumors and metastatic cancer sites that are unreachable by EPR targeting is by cell-mediated delivery of nanoparticles. Conjugating nanocapsules encapsulating the topoisomerase I drug SN-38 to the T cell surface, used to traffic through the lymphatic system, resulted in a 90-fold increase of SN-38 drugs in lymph nodes relative to free drug [168]. Immune cell-induced delivery of nanoparticles can improve drug accumulation in disseminated tumors and metastasis. Such a safer targeted delivery of IFN-γ can promote the differentiation of tumor-enhancing M2 macrophages to antitumor M1 [21].

To better clinical results, patients showing a sufficient level of EPR may be pre-selected. Suitable, efficient probes and protocols are necessary for patient pre-selection for clinical trials in considering factors, including vascular leakiness and perfusion, macrophage content, and density of ECM. To quantify the EPR effect in tumor targeting, direct and indirect imaging techniques have been promising. MRI scanning was used to characterize and correlate parameters including RBV and vessel permeability with the accumulation of fluorophore-labeled nanoparticles in several tumor models to detect biomarkers of EPR-mediated drug accumulation that have been studied [169]. The accumulation of polystyrene nanoparticles in different tumor models has been evaluated using multi-modal imaging techniques [170].

The PET nano-reporter, can serve as a companion nano-diagnostic for Doxil, loaded with chelators allowing for ^89^Zr-labeling and PET imaging to evaluate the therapeutic outcome by predicting the drug accumulation. Nano-reporter and doxorubicin concentrations in tumors correlated well with therapeutic efficiency, indicating a good therapeutic response [171]. A paramagnetic, ferumoxytol, was studied as a companion nano-diagnostic with polymeric nanoparticles encapsulating docataxel, to differentiate tumors with high, medium and low accumulation of docetaxel and ferumoxytol. The highest docetaxel accumulation and tumor response was observed for high ferumoxytol accumulated tumors [20].

^64^Cu-labelled, HER2-targeted PEGylated liposomes containing doxorubicin were used to study the EPR effect in primary and metastatic breast cancers by quantitative PET imaging and biopsies to determine the doxorubicin accumulation in the tumor and therapeutic efficacy [172].

The various direct and indirect imaging strategies discussed above for EPR determination have their own advantages and difficulties. However, the use of indirect imaging is promising for EPR measurement and patient pre-selection for therapy. The clinically approved and imageable companion diagnostic, ferumoxytol, has shown potential in patient pre-selection, evaluation of EPR effect, and improvement of EPR-based tumor targeting [20].

The typical xenograft mouse model uses inoculation of simple human cell lines and immuno-compromised mice. Nanodrug accumulation is lower in immuno-deficient mice compared to immuno-competent mice. These mouse models are highly homogeneous compared to the patient tumor with a typically low degree of heterogeneity. Tumors in mice usually have a relatively large size. However, compared to the size of the tumor in patients, they are usually relatively small. The murine tumors also lack the patient’s TME and stromal factors due to their very different growth kinetics, usually days and weeks in mice, when contrasted with months and even years in patients. Metastasis is often ignored in xenograft models. With EPR-mediated drug delivery, tumor location plays a crucial role, with a tendency for higher accumulation in orthotopic tumors. Age differences between human and mouse may further affect the EPR, as mice at a very young age are usually selected for tumor inoculation and tumors grow rapidly However, human tumors, at old age, may grow slowly and progress only over several years [5,20].

Organoids and PDX models are very attractive for preclinical research because of the regrowth of human tumors. The tumor cells are harvested through biopsy or surgery. Upon growth in PDX model or organoids, most of the tumor stroma and TME features, and structure are retained. PDX models facilitate the study of several parameters, including TME and vascularization of the tumor and accumulation of drugs indicating the extent of EPR. However, there still exist limitations for translating PDX and organoids study benefits to clinic, since PDX models depend on immuno-compromised mice and organoids and PDX models require time and labor-intensive workflow with low engraftment rates.

## 8. The EPR Effect and Beyond: Enhancement of Therapeutic Efficacy of Cancer Nanomedicine

EPR-mediated drug systems have prodigious therapeutic potential. However, intra- and inter-tumoral, inter-patient heterogeneity in EPR effect, and physiological barriers associated with it pose a big challenge in drug delivery. Actively targeted drug delivery systems are greatly beneficial for the treatment of hematological malignancies. For solid tumors, active targeting must rely on EPR-mediated accumulation in tumors. To assess the EPR effect, the development of fast, quantitative EPR-imaging technologies is necessary. Several strategies have also been proposed that bypass the EPR effect, including cell-mediated delivery of nanoparticles and immune-modulating payload release in tumors. Such approaches should be useful for the treatment of low-EPR tumors, hematological malignancies, and metastatic cancers. Besides, local delivery of drugs using nanoparticles, hydrogels, implants, etc., can bypass physiological barriers of EPR-targeted delivery [5,20,21]. Moreover, the CAST therapy might be another approach to compensate for the inadequate effect of the EPR targeting [14]. Recently, mAb-based therapies for solid tumors has been proposed to overcome tumor heterogeneity, efficient delivery to tumor, durable therapeutic and clinical outcome in a large subset of patients, and enhanced prognosis [18]. Table 3 shows some selected EPR-based drug delivery systems [173].

**Scheme 1:**
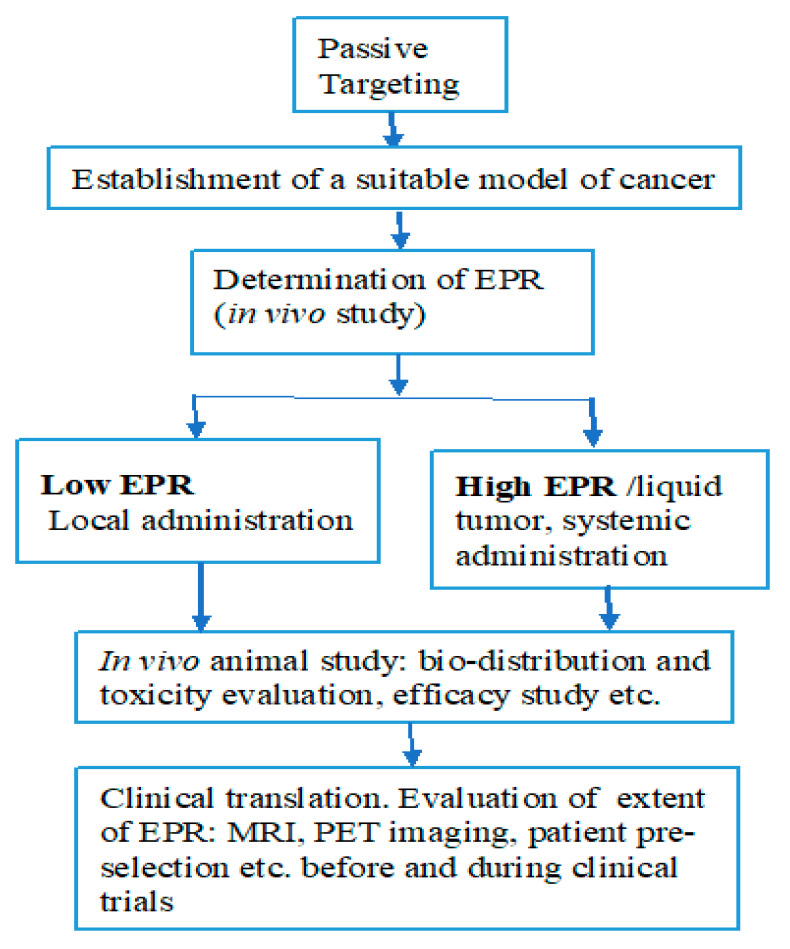
Proposed workflow in the development of an EPR-based drug delivery system.

Development of more predictive animal models, including PDX and organoids, and EPR-imaging techniques and adoption of GLP, standardization guidelines, are necessary for translation of ERP-based therapies to the clinic. Development of PDX and organoid libraries to share information of the EPR patients, EPR tumors, and outcome of EPR-mediated drug delivery research will be helpful to predict appropriate therapy for the patients more rapidly and accurately. Scheme 1 shows the proposed workflow in the development of EPR-based drug delivery systems.

Active targeting can also be used as a complementary approach to EPR-mediated tumor targeting to enhance drug delivery, accumulation, and retention in tumors [174]. Finally, pharmacological and physical co-treatments, nanoparticle-based combination therapies, bio-inspired design of nanoparticles allowing tumor-selective drug release, advanced imaging techniques coupled with HTP computing technologies, and development of 3D-models of cells, better animal models, and organoids are necessary to improve the application and efficacy of EPR-targeted drug delivery for cancer therapy in both clinical and pre-clinical settings.

## 9. Conclusions and Future Perspective

EPR-targeted drug delivery was first discovered by Maeda and colleagues in solid murine tumors and reached the clinic about 30 years ago with the approval of the first EPR-based drug. Interpersonal and intra-, as well as inter-tumoral heterogeneity, is a major difficulty in EPR drug delivery studies. Since drug accumulation through EPR effect in the tumor may not be enough to obtain therapeutic effect, penetration, internalization, effective drug delivery, and cytoplasmic release are crucial for improvements with anticancer therapy. Actively targeted drug delivery systems have been highly useful for the treatment of hematological malignancies, whereas, for solid tumors, active targeting must rely on EPR-mediated accumulation in tumors. Quantification of the EPR-effect is thus necessary. To assess the EPR effect, the development of fast, quantitative EPR-imaging technologies is essential. Combination therapeutic approaches using nanoparticles, complementary use of passive and active targeting, and pharmaceutical as well physical co-treatments are crucial for enhancing EPR-based drug delivery systems. Humanized advanced animal models, 3D-models and organoids development, and patient pre-selection may improve EPR-based tumor targeting and clinical translation.

## Figures and Tables

**Table 1:** Properties of nanoparticles that affect the EPR.

	Properties of Nanoparticles		
	Size	Charge	Shape
Characteristics	It is evident from the mouse xenograft models that smaller molecules that are 3.3 to 10 kDa with 2 and 3 nm diffused deeper into the tumor compared to the large molecules [59]. Nanoparticles smaller than 70 nm tend to aggregate in the tumor if they are highly permeable [60].	Surface charge of the nanoparticles plays a key role in the clearance of the nanoparticles and thereby the residence time in the body. It also changes the opsonization profile of the nanoparticle [61]. An increased accumulation in the tumor site has been observed for nanoparticles that are cationic and sterically stabilized [62]. Both positively and negatively charged nanoparticles have an affinity to bind to the components in the extracellular matrix, thereby decreasing the diffusion in the interstitium [19].	Increased accumulation is observed in the nanoparticles that are elongated, such as carbon nanotubes with a high aspect ratio (100:1 to 500:1). Porous media can aid in the filtration process [63]. Nanorods 44 nm in length have been shown to extravasate more than 35 nm length rods by four-fold [64].

**Table 2:** Recent progress and status of patient-derived tumor models.

Therapeutic Moiety/Combination	Target	Cancer Type	Animal Type	Reference
Erlotinib	EGFR	Glioblastoma	Athymic nude mice	[100]
Gefitinib and Enzalutamide	Androgen receptor and EGFR	non-small cell lung cancer and Prostate cancer	Chick chorioallantoic membrane (CAM)	[101]
Apatinib, Regorafenib, Cabozantinib, Ramucirumab	VEGFR2	Gastric cancers	Zebrafish	[102]
Ramucirumab	Her2, FGFR2, cMet	Gastric cancers	BALB/c nude mice	[103]
Bortezomib	CDK4 and MDM2	Liposarcoma	Mice	[104]
Pembrolizumab	PD-1/PD-L1	Soft Tissue Sarcoma	NSG mice	[105]
Erdafitinib	FGFR	Metastatic prostate cancer	Male mice	[106]
β-elemene and cisplatin-coloaded liposomes	Codelivery to reverse MDR	Lung cancer	C_57_BL/6 mice	[107]

**Table 3:** Selected EPR -based therapeutic systems.

Carrier	Ligand	Imaging/Therapeutic Agent	Applications
Nanoemulsion	PEGylated hydrophilic molecules (Killiphore ELP)	Iodinated monoglyceride and iodinated castor oil contrast agent	Blood pool imaging agents, accumulated particularly in liver and spleen and imaged by X-ray, CT.
Albumin nps	-	Tacrolimus (TAC)	TAC-loaded HAS nps, target inflamed joints of rheumatoid arthritis tissues
Polymeric nps	C18PMH-PEG	Fe_3_O_4_ contarst agents and doxorubicin drug	Magnetically controlled drug delivery and for T2-weighted MRI imaging
Lipid nanocapsules	Polysaccharide lipochitosan and liopdextran	DiD fluorescent dye	Selective to HEK293 (β3) cells bearing mice, detected by imaging
